# Time for cardiac care in pregnancy: beyond 42 days post-partum

**DOI:** 10.21542/gcsp.2021.6

**Published:** 2021-04-30

**Authors:** Karen Sliwa, Ana Mocumbi

**Affiliations:** 1 Cape Heart Institute, Faculty of Health Sciences, University of Cape Town, Chris Barnard Building, Cape Town, South Africa; 2 Instituto Nacional de Saúde, Marracuene, Moçambique; 3 Faculdade de Medicina, Universidade Eduardo Mondlane, Maputo, Moçambique

Avoidable deaths from pregnancy complications occur on a global scale, with the greatest burden of mortality among women in low-to-middle income countries^[1]^. Most countries record maternal death only up to 42 days postpartum because of the assumption that avoidable death in pregnant women occurs during pregnancy or shortly thereafter. Although limited, the available data suggest otherwise.

The WHO Working Group on Maternal Mortality (WHO-International Classification Disease 2013)^[3]^ has suggested International Classification of Diseases (ICD) coding principles that define maternal death up to a year after delivery from causes directly related to pregnancy or indirectly precipitated by the effects of pregnancy on underlying diseases. The ICD Code (ICD10) makes it obligatory to document the occurrence of pregnancy within a year of the death of any woman. What is known as late maternal death falls into the main following categories: cardiovascular cause, thromboembolism, cancer, and suicide (often related to postpartum depression).

Globally, there are more postpartum and late maternal deaths from direct and indirect obstetric causes than maternal deaths during pregnancy^[1]^. Maternal mortality, no matter when and where it occurs, results in sequelae that extend beyond the loss of the life of a single woman. The death of a mother adversely affects the ability of her family to survive and thrive, especially under conditions of socioeconomic deprivation. The overall poor documentation of late maternal death is a neglected global responsibility^[2]^.

The article by Suzy Kotit and Magdi Yacoub on *Cardiovascular adverse events in pregnancy: A global perspective* (https//doi.org/10.21542/gcsp.2021.5) a global perspective of cardiovascular maternal mortality, focusing on events during and after pregnancy, their predictors and risk stratification.

First, the authors address the disparity in maternal mortality rates in the different regions of the world, and discuss the tools available to estimate morbidity and mortality risk in pregnant women with cardiac disease. Additionally, they discuss the continuum of maternal risk from pregnancy to late after childbirth, and highlight the mounting evidence pointing to a significant link between several events and the risk of cardiovascular disease later in life. Finally, they suggest the ways forward towards reduction of preventable maternal deaths due to cardiovascular disease.

Pregnancy can trigger cardiovascular disease (e.g., hypertensive disorders leading to heart failure), aggravate underlying disease (e.g., congenital heart disease, rheumatic heart disease, or pulmonary arterial hypertension), or cause specific diseases, such as peripartum cardiomyopathy (PPCM).

PPCM is a global disease that is often not diagnosed timely, leading to significant morbidity and mortality. It is an important contributor to early (<42 days) and late (up to 1 year) postpartum maternal death. The reported 1-year mortality ranges from 5–25% ^[4,5]^.

The fact that many of the women with cardiac disease in the peripartum period are diagnosed beyond the standard date of reporting (<42 days) is of concern because no matter how late these deaths occur, they are related to pregnancy. Additionally, counselling about the risks of future pregnancy, access to adequate contraceptive services, and adequate therapeutic management is not always provided^[6]^. Figure 1 summarizes key parameters that impact on the prognosis of peripartum women presenting with cardiovascular disease that goes beyond the clinical condition but highlights the socioeconomic parameters.

**Figure 1.**
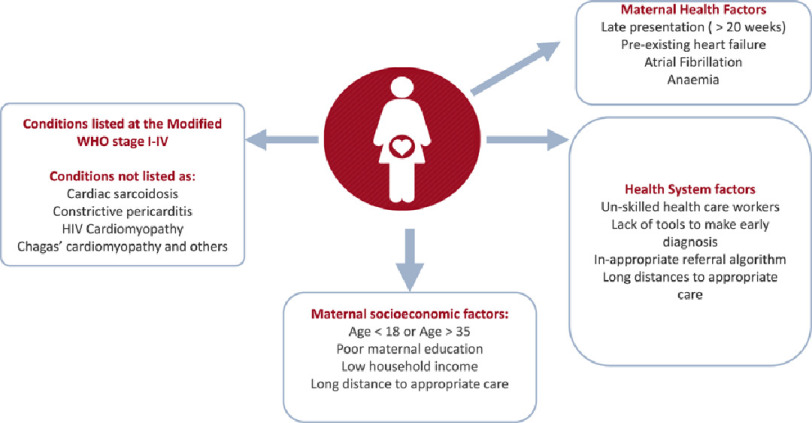
Factors contributing to increased maternal and fetal risk in pregnant women with heart disease.

In the last two decades, remarkable advances in the comprehension of the pathogenesis and the improvement in peripartum patient management and therapy have been achieved, largely due to team-efforts and close collaboration between basic scientists, cardiologists, intensive care specialists and obstetricians.

There is substantial progress in the options of cardiovascular imaging modalities available for pregnant women to confirm the diagnosis, to assess disease severity and stratify risk, to prognosticate, to plan for appropriate management and to assess response to therapy.

There are many examples of recent studies on cardiac condition in the peripartum period^[7]^. Under the umbrella of the Heart Failure Association of the European Society of Cardiology (ESC), a PPCM Study Group was established in 2009, which led to several research projects advancing the knowledge about this condition^[8]^.

The need to create more awareness about this condition globally, as well as to understand differences in mode and presentation, led to the establishment of the international registry on PPCM, funded by the ESC under the umbrella of the *EuroObservational research program,* which recruited >700 patients from more than 40 countries. Follow-up will be concluded in 2021. Many lessons will be learned from this program but also from the multi-centre Nigerian PEACE PPCM registry^[9]^ and the large clinical service dedicated to women with PPCM in Iraq ^[10]^.

We hope that over the next decade many more studies on peripartum women with cardiac conditions will be conducted in low-to-middle income countries to not only create better awareness but to improve patient care.

## Conflicts of interest:

Nothing to declare related to the content of this editorial.
